# Facile preparation and adsorption performance of low-cost MOF@cotton fibre composite for uranium removal

**DOI:** 10.1038/s41598-020-76173-4

**Published:** 2020-11-06

**Authors:** Aili Yang, Zhijun Wang, Yukuan Zhu

**Affiliations:** Institute of Materials, China Academy of Engineering Physics, Jiangyou Sichuan, 621907 China

**Keywords:** Pollution remediation, Nuclear chemistry

## Abstract

A novel composite MOF@cotton fibre (HCF) was prepared and characterized by FTIR, SEM, XPS and TGA. The effect of various parameters on the adsorption efficiency, such as the solution pH, contact time, initial U(VI) concentration and temperature, was studied. The maximal sorption capacity (*Q*_m_) is 241.28 mg g^−1^ at pH 3.0 for U(VI) according to the Langmuir isotherm adsorption model, and the kinetic and thermodynamic data reveal a relatively fast entropy-driven process (Δ*H*^0^ = 13.47 kJ mol^−1^ and Δ*S*^0^ = 75.47 J K^−1^ mol^−1^). The removal efficiency of U(VI) by HCF is comparable with that of pure cotton fibre and as-prepared MOF (noted as HST). However, the HST composite with cotton fibre significantly improved the treatment process of U(VI) from aqueous solutions in view of higher removal efficiency, lower cost and faster solid–liquid separation. Recycling experiments showed that HCF can be used up to five times with less than 10% efficiency loss.

Uranium (U) is a key radioactive element for nuclear fuel in the nuclear reactors. The remarkable development of nuclear activities related to U such as mining, refinement and recovery of U is of critical importance for the continued development of nuclear energy^1,2^. To decrease the release of U into the environment and contamination risks in case of incidents or nuclear events, an adsorption approach based on various high-efficiency sorbents has been proposed and attracted considerable attentions^3–7^. Adsorption has been examined as an alternative method to remove U from aqueous solutions due to its simple operation, low cost, energy saving, high efficiency, etc Cellulose and their derivatives have achieved extensive attention due to their advantages such as the most abundant resource, biocompatibility, inexpensiveness and eco-friendliness^8,9^. However, pure cellulose generally exhibits poor capacity in practical applications and must be chemically modified^10–12^. The composite of cellulose with other substances is an efficient approach to enhance their performance and extend their applications^13–16^.

As a class of important porous materials, metal–organic frameworks (MOFs) have outstanding characteristics including extra-high surface areas, diverse functionalities, linkers tailorability, acid and base robustness and tunable aperture^17,18^. Thus, they have great potentials in diverse domains such as gas storage and separation^19^, sensing^20^, adsorption^21–23^, biomedicine^24^, magnetism^25^, luminescence^26^, environmental remediation^27–29^, and catalysis^30,31^. MOFs@cellulose composites have been proven to provide better benefits for their versatile applications than pure cellulose or MOFs^32^. However, most of the recent achievements have highlighted the antibacterial properties^33–37^, gas adsorption^38^ and pollutant removal^39–42^ of MOF@cellulose composites. To our best knowledge, the integration of HKUST-1 with cellulose to synergistically enhance their intrinsic prominent properties for the removal of U(VI) has never been reported, except for a few reports on the removal of U(VI) using cellulose derivatives^43–52^. In addition, the production cost of cellulose derivatives must be significantly reduced to further improve their real applications. Our research has the following contributions: (i) a novel, facile and low-cost method to prepare MOF@cotton fibre (HCF) composite, which uses a much cheaper benzoic acid (BA; the cost of the analytical-grade product is 16.4 ¥/250 g) as the ligand to replace part of the traditional ligand trimesic acid (H_3_BTC to synthesize HKUST-1; the cost of the analytical-grade product is 566 ¥/500 g); (ii) fibriform HCF, which is very easily separated from the liquid phase; (iii) favourable adsorption efficiency for U(VI), where the maximal adsorption capacity is 241.28 mg g^−1^.

In the present study, a novel MOF@cotton fibre (HCF) composite was facilely synthesized by a simple hydrothermal method. To characterize the obtained materials, X-ray photoelectron spectroscopy (XPS), FT-IR spectroscopy (FTIR), scanning electron microscopy (SEM) and thermogravimetric analysis (TGA) were used. The effects of a few critical parameters (pH value, contact time, initial U(VI) concentration and temperature) and the adsorption kinetics, adsorption isotherms and reusability of HCF were investigated in detail.

## Results and discussion

### Characterization

**Figure 1:**
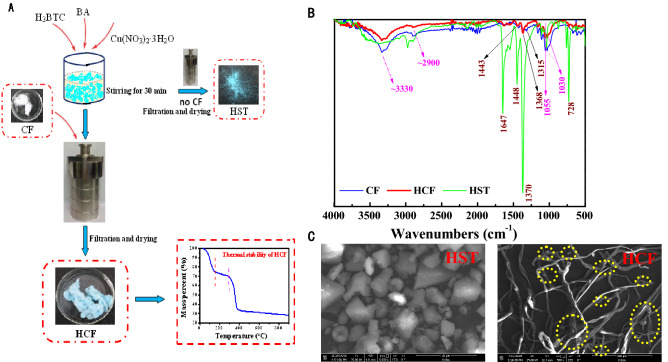
(**A**) Preparation flow diagram and thermal stability of HCF and morphology characterization; (**B**) FTIR spectra of the samples; (**C**) SEM images of HST (× 5000) and HCF (× 500).

The preparation flow diagram and FTIR and SEM image characterization of the samples are shown in Fig. 1. To reduce the preparation cost of the adsorbent, BA with lower cost was used as the ligand to replace part of the traditional ligand H_3_BTC. The blue powder product HST was obtained by a simple solvothermal method. While we added CF into the HST precursor (Cu^II^, BA and H_3_BTC) solution for the solvothermal synthesis, the blue fibrous product HCF was fabricated (see Fig. 1A). The thermal stability of HCF was shown in Fig. 1A.

The functional group structures of the samples were characterized by FTIR spectra as shown in Fig. 1B. The characteristic peaks at 1370 cm^–1^, 1448 cm^–1^ and 1647 cm^–1^ were attributed to C–O, C=O and aromatic C=C of H_3_BTC, respectively^53^. The characteristic peaks at 1368 cm^–1^ and 1443 cm^–1^ appeared in the FTIR of HCF. Similar characteristic peaks at ~ 3330 cm^–1^, ~ 2900 cm^–1^, 1055 cm^–1^ and 1030 cm^–1^ appeared in the IR of both HCF and CF. These results show that HST combined with CF successfully to produce HCF.

**Figure 2:**
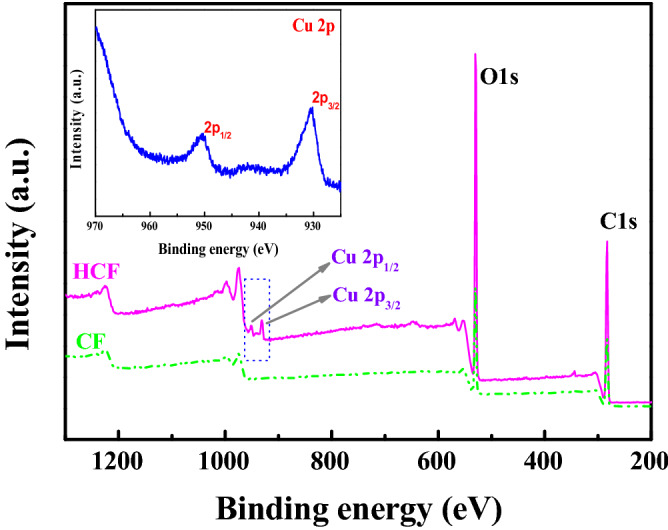
XPS survey spectra of HCF and CF. Insert: high-resolution Cu 2p spectra of HCF.

The surface morphologies of HST and HCF observed in SEM images are shown in Fig. 1C. The as-synthesized HST particles with octahedral and spherical shape (highlighted as yellow dashed circles) were distinctly observed in the surfaces of CF. XPS was used to study the composition and valence of HCF and CF. Figure 2 shows the XPS survey spectra of HCF and CF. In Fig. 2 and the insert, two main peaks at 930.2 and 952.0 eV, which are ascribed to Cu 2p_3/2_ and Cu 2p_1/2_ signals, correspond to the characteristic peak of Cu^2+^ in the XPS survey spectrum of HCF, which indicates the presence of HST in the composite HCF^54^.

### Effect of the pH and ion strength on the U removal

**Figure 3:**
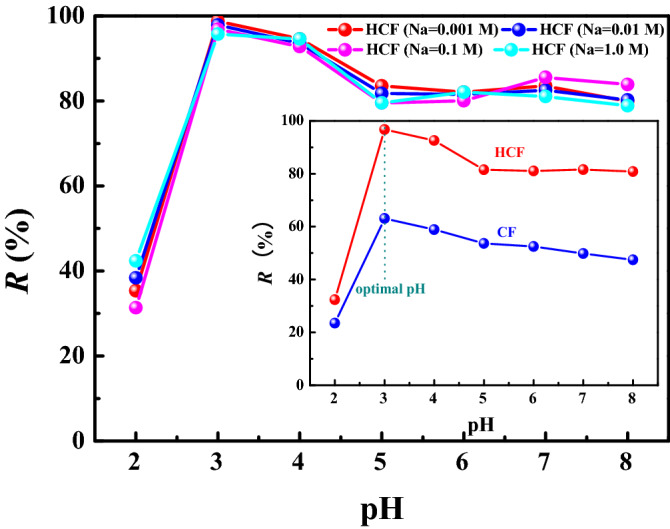
Effect of the ion strength on the removal rate. Insert: effect of pH on the removal rate (pH = 3.0, m/V = 0. 5 g L^−1^, *C*_0_ = 10 mg L^−1^ and contact time = 30 min).

The chemistry species of uranium in the solutions significantly varies at different pH, which makes the solution pH greatly affect the adsorption efficiency. The effect of the solution pH of 2–8 and ion strength with different concentrations of NaClO_4_ (0.001, 0.01, 0.1 and 1.0 M) on the uranium adsorption is shown in Fig. 3. In Fig. 3 (insert), the removal rate of U(VI) by HCF and CF was maximal at pH = 3. It is well known that the most predominant uranium species is UO_2_^2+^ at low pH; therefore, the removal rate was low due to the competition between abundant H^+^ and UO_2_^2+^ ions^55^. With increasing solution pH when U is mainly present as negatively charged species (UO_2_)_3_(OH)_7_^-^ and (UO_2_)(OH)_3_^-^, the removal efficiency increased due to the electrostatic attraction between the negatively charged U(VI) species and the positively charged sorbent^56^. When the pH further increased, the hydrolysation of UO_2_^2+^ and formation of U(VI)-carbonate species (UO_2_)_2_(CO_3_)(OH)^3−^ inhibited the U(VI) adsorption, which decreased the adsorption capacity^57^. Therefore, an optimal pH of 3.0 was used in subsequent experiments. In addition, the ion strength clearly had little effect on the adsorption process, which suggests that the inner-sphere surface complexation mechanism plays a main role in the adsorption process^58^.

### Effect of the contact time and kinetic studies

**Figure 4:**
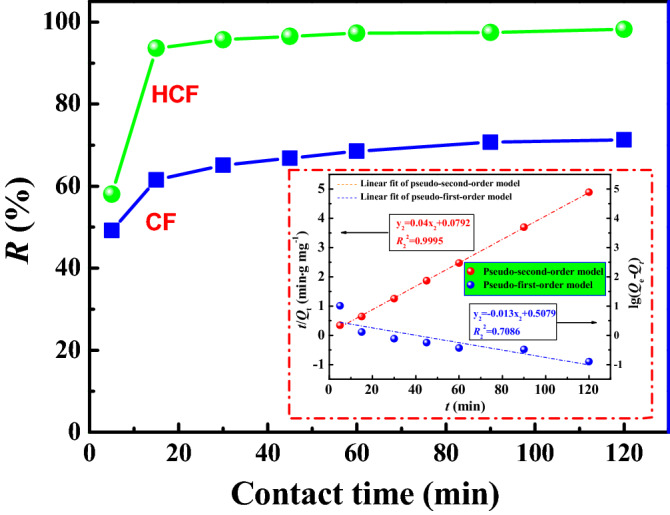
Effect of the contact time on the U(VI) sorption (pH = 3.0, *C*_(U)initial_ = 10 mg L^−1^ and *m/V* = 0.4 g L^−1^). Insert: linear fit of the pseudo-first-order and pseudo-second-order kinetics models.

The adsorption efficiency of the adsorbents can be evaluated by the adsorption equilibrium time and kinetic process. Figure 4 presents the effect of the contact time of 5–120 min on the U(VI) sorption by HCF and CF in regard to the kinetics at an initial U(VI) concentration of 10 mg L^−1^ at pH 3.0 at room temperature. With the extension of time, the adsorption efficiency significantly increased until it reached an equilibrium within 30 min. The removal rate of HCF for U(VI) was nearly 100% within 30 min, while the removal efficiency of CF was poor (only approximately 70%). Pseudo-first-order and pseudo-second-order models were used to study the adsorption kinetics of U(VI) on HCF. Figure 4 (insert) clearly shows that the pseudo-second-order model had a superior correlation coefficient (*R*^2^) compared to the pseudo-first-order model, which indicates that the U(VI) adsorption processes of HCF well fit the pseudo-second-order model.

### Adsorption isotherms and thermodynamic studies

**Figure 5:**
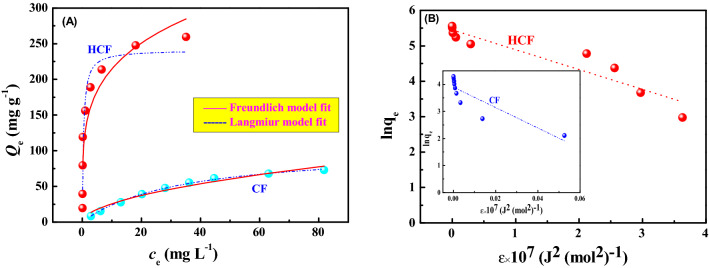
Langmuir, Freundlich model fit (**A**) and D–R isotherm plots (**B**) of U(VI) onto HCF and CF (pH = 3.0, *C*_(U)initial_ = 5–150 mg L^−1^, *m/V* = 0.4 g L^−1^ and contact time = 24 h).

Langmuir and Freundlich adsorption isotherm models are expressed in Eqs. (1) and (2), respectively^59^. According to Eqs. (1) and (2), the maximal adsorption capacity (*Q*_m_) was fitted when the initial U(VI) concentration was 5–150 mg L^−1^ (Fig. 5). The calculated Langmuir and Freundlich isotherm parameters from the fitting processes are listed in Table 1. Table 1 shows that Langmuir isotherm fitted the experimental data well with a higher correlation coefficient (*R*^2^), and the maximum adsorption capacity was 241.28 mg g^−1^ for the adsorption of HCF. Moreover, Fig. 5 shows that HCF is a promising sorbent for the removal of uranium from aqueous solutions in terms of the preparation cost, adsorption efficiency and simple solid–liquid separation for uranium from aqueous solutions.

1 $$Q_{e} = \frac{{Q_{m} \cdot K_{L} \cdot C_{e} }}{{1 + K_{L} C_{e} }},$$

2 $$Q_{e} = K_{F} \cdot C_{e}^{1/n} ,$$

where *Q*_*e*_ (mg g^−1^) is the equilibrium adsorption capacity; *C*_e_ (mg L^−1^) is the uranium concentration at equilibrium; *Q*_*m*_ (mg g^−1^) is the maximum adsorption capacity; *K*_L_ (L mg^−1^) and *K*_F_ (mg^1−n^ L^n^ g^−1^) are Langmuir constant and Freundlich constant, respectively; *n* is Freundlich adsorption exponent.

**Table 1:** Langmuir, Freundlich and D–R isotherm model parameters for U(VI) adsorption onto HCF and CF.

Sorbents	Langmuir			Freundlich			D-R			
	*Q*_m_ mg g^−1^	*k*_L_ L mg^−1^	*R*^*2*^	*n*	*k*_F_ mg^1−n^ L^n^ g^−1^	*R*^2^	q_s_ mg g^−1^	*β* mol^2^ (J^2^)^−1^	*E* kJ mol^−1^	*R*^2^
HCF	241.28	2.418	0.9152	4.45	127.91	0.8466	232.93	0.56	0.56	0.8724
CF	104.56	0.029	0.9976	1.89	7.6110	0.9629	49.34	37.81	0.12	0.7363

Dubinin–Radushkevich (D–R) isotherm is usually employed to explain the adsorption mechanism with respect to Gaussian energy distribution onto a heterogeneous surface and determine the adsorption nature as physical or chemical based on the mean free energy (*E*)^60^. The D–R isotherm model is expressed by Eq. (3). Model parameters *ε*, *β* and *E* can be determined and calculated by Eqs. (4) and (5).

3 $$\ln Q_{e} = \ln Q_{m} - \beta \varepsilon^{2} ,$$

4 $$\varepsilon = RT\ln \left( {1 + 1/C_{e} } \right),$$

5 $$E = \frac{1}{{\sqrt {2\beta } }},$$

where *β* (mol^2^ (J^2^)^−1^) is D–R isotherm constant, and *ε* (J mol^−1^) is Polanyi potential. The calculated D–R isotherm parameters are listed in Table 1. From the obtained *E* value, the physical or chemical sorption mechanism can be revealed. According to the literature^61^, if *E* is 8–16 kJ mol^−1^, the sorption process chemically occurs, whereas *E* < 8 kJ mol^−1^ follows the physical sorption. For HCF, the low *E* value of 0.56 kJ mol^−1^ in this study suggests that the U adsorption was a physical adsorption process due to electrostatic or Van der Waal’s attractions.

The linear form of the Toth equation was as following^62^. A linear relationship can be obtained by plotting (*c*_e_/*q*_e_)^*T*^ against (*c*_e_)^*T*^ at different *T* values, and then the values of *q*_*T*_, *b*_*T*_ and *R*^2^ can be calculated.

6 $$\left( {\frac{{c_{e} }}{{q_{e} }}} \right)^{T} = \frac{1}{{(q_{T} b_{T} )^{T} }} + \frac{1}{{q_{T}^{T} }}c_{e}^{T} ,$$

where *q*_*T*_ (mg g^−1^) and *b*_*T*_ (L mg^−1^) are the parameters of the Toth equation, *T* is exponent of the Toth equation.

**Figure 6:**
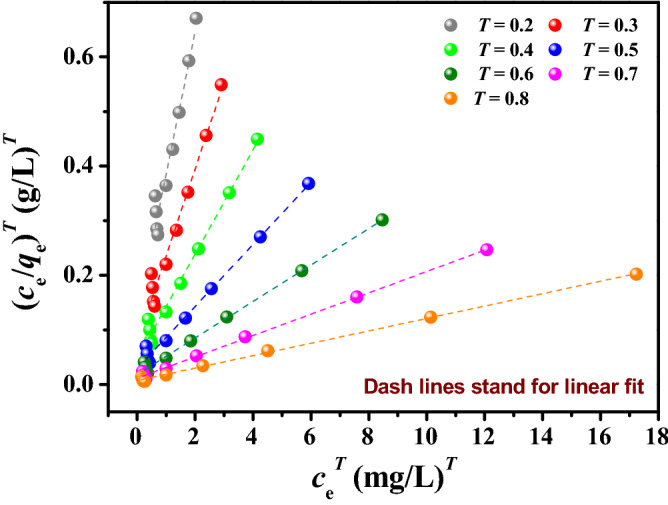
Linear fit of the Toth equation at different *T* for the adsorption of uranium on HCF.

Figure 6 shows the regression results for the adsorption of uranium on HCF. The best regression line is identified when *T* is above 0.8 and the *R*^2^ value of the line is closest to 1. The calculated values of *q*_*T*_, *b*_*T*_ and *R*^2^ are also listed in Table 2. Table 2 indicates that the values of *R*^2^ increase with the increase of the values of *T*. As a result, the Toth equation (*T* > *0.8*) reveals better-fitting results than the Langmuir, Freundlich and D–R equations. According to the reference^63^, *T* is 0.6–1.0 for the adsorption of uranium, meaning that the adsorption occurs mostly on homogeneous surfaces in this study.

**Table 2:** Parameters of the Toth equations in the adsorption of uranium on HCF.

*T*	Parameters of the Toth equations		
	*q*_*T*_ (mg/g)	*b*_*T*_ (L/mg)	*R*^2^
0.2	841.65	40.53	0.9548
0.3	468.97	11.47	0.9718
0.4	359.49	5.81	0.9832
0.5	318.88	3.73	0.9903
0.6	288.69	2.70	0.9944
0.7	277.21	2.10	0.9966
0.8	269.64	1.71	0.9977

A comparison of the maximum adsorption capacity (*Q*_m_) of various adsorbents is shown in Table 3. According to Table 3, the as-synthesized HCF clearly presents higher *Q*_m_ than other cellulose-based adsorbents. This increased adsorption capacity of HCF for U(VI) may be attributed to its fibrous structure and the addition of HST with higher specific surface area and excellent adsorption efficiency^64^ in the surface of CF. *Q*_m_ of the as-prepared HCF shows that HCF is a promising adsorbent for the treatment of uranium-bearing wastewater.

**Table 3:** Maximum adsorption capacity (*Q*_m_) of cellulose-based adsorbents for U(VI) ions.

Cellulose-based sorbents	pH	*m/V* (g L^−1^)	*Q*_m_ (mg g^−1^)	References
Amine-impregnated cellulose	0.1–3.0	2.5	54.5	^43^
Urea-cellulose	0.1–3.0	2.5	82	^44^
CMC-Al	5.0	–	12.1	^45^
HPMC-g-AO film	4.1	–	765	^48^
Polyacrylonitrile fibres	5.0	1.0	163	^6^
Graphene oxide-cellulose	5.0	1.0	101.01	^66^
CMC/MGOs	5.5	0.25	188.97	^67^
CSP-CMCP	5.0	0.05	977.54	^68^
Arg-Cell, Glu-Cell	5.0	0.4	147, 168	^69^
SA/CMC-Ca-Al	4.0	–	101.76	^70^
CMC-FeS	5.0	–	430.3	^71^
P(IA/MAA)-g-NC/NB	5.5	2.0	119.63	^72^
CMC-INP	5.0	–	322.58	^73^
HCF	3.0	0.4	241.28	This study

**Figure 7:**
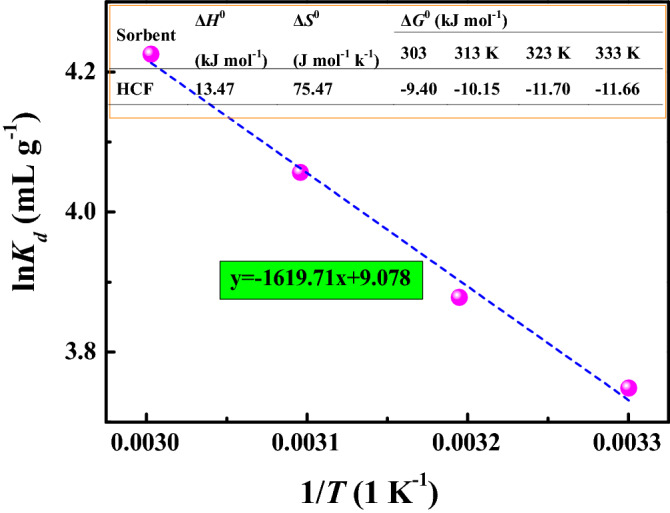
ln*K*_*d*_ versus 1/*T* for U(VI) adsorption onto HCF. pH = 3.0, *C*_(U)initial_ = 10 mg L^−1^, *C*_sorbent_ = 0.4 g L^−1^, *T* = 303 K, 313 K, 323 K and 333 K, and contact time = 24 h.

To evaluate the adsorption thermodynamic parameters, the effect of temperature on the uranium removal was investigated using 20 mL solutions containing 0.008 g of HCF and 10 mg L^−1^ U(VI), which was shaken for 24 h at pH 3.0. After the adsorption equilibrium, adsorption results of U(VI) by HCF were obtained, and the plots of ln*K*_d_ versus 1/*T* onto HCF is shown in Fig. 7. Thermodynamic parameters (i.e., enthalpy (Δ*H*^0^), entropy (Δ*S*^0^) and standard free energy Δ*G*^0^) from 303 to 333 K in the adsorption process were calculated from the slope and intercept of the linear line of ln*K*_*d*_ versus 1/*T* according to Vander Hoff Eqs. (7) ~ (9)^65^. The evaluated Δ*H*^0^ value is 13.47 kJ mol^−1^, which reflects that the adsorption reaction was endothermic. The obtained positive Δ*S*^0^ and negative Δ*G*^0^ values (Fig. 7 insert) suggest that the adsorption process was spontaneous.

7 $$K_{d} = \frac{{c_{ad} }}{{c{}_{e}}},$$

8 $$\ln K_{d} = - \frac{{\Delta H^{0} }}{RT} + \frac{{\Delta S^{0} }}{R},$$

9 $$\Delta G^{0} = \, \Delta H^{0} - T\Delta S^{0} ,$$

where *K*_*d*_ (mL g^−1^) is the distribution coefficient of U(VI); *c*_ad_ (mg L^−1^) is the concentration of metal ions on the adsorbent at equilibrium; *c*_e_ (mg L^−1^) is the equilibrium concentration of metal ions in solution; *R* (8.314 J mol^−1^ K^−1^) is the universal gas constant; *T* (K) is the absolute temperature.

### Regeneration and reuse of HCF

**Figure 8:**
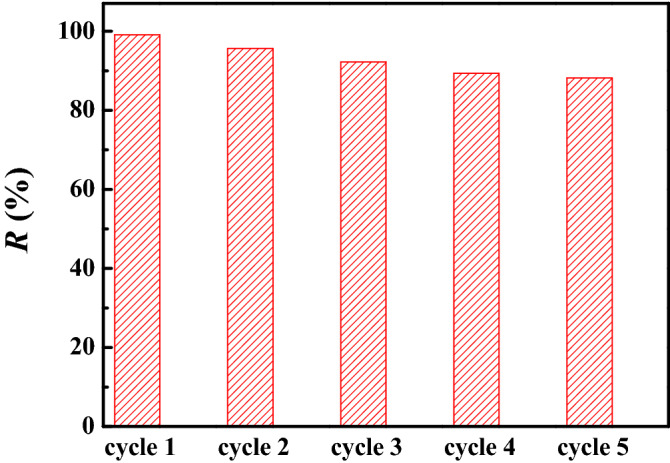
Regeneration and reuse studies of HCF (pH = 3.0, *C*_(U)initial_ = 10 mg L^−1^, *m/V* = 0.40 g L^−1^ and contact time = 24 h).

The adsorbent reuse is an important index to further evaluate their adsorption performance and reduce the treatment cost. After adsorption and filtration, the adsorbent loaded U(VI) was collected and treated with excess HNO_3_ (0.1 mol L^−1^) for 24 h in a shaker. Then, the adsorbent was separated from the liquid by filtration and washed several times by DW to be used for the next cycle adsorption experiment. The reuse experiments of HCF were performed for five cycles. According to Fig. 8, the U(VI) removal percentage with HCF was 99.11% after the first cycling experiment. After five recycling experiments, the U(VI) removal percentage remained at 88.24%, which suggests that HCF can be recovered and reused several times and has favourable recycling capability.

## Materials and methods

### Materials

The cotton fibre (CF) in this study was medical purified cotton from Xvzhou hygienic materials plant (Jiangsu, China). Copper(II) nitrate trihydrate (Cu(NO_3_)_2_·3H_2_O) was provided by Chengdu Kelong Chemical Co., Ltd. Benzoic acid (BA), and trimesic acid (H_3_BTC) was purchased from Aladdin Chemical Reagent Co., Ltd. All reagents were of analytical grade and used without further purification. UO_2_(NO_3_)_2_·6H_2_O was provided by Xi’an Dingtian Chemical Reagent Co., Ltd. The deionized water (DW) was used throughout the experiments.

### Synthesis

HCF was synthesized through a simple solvothermal method. In a typical synthesis of HCF, 1.0870 g of Cu(NO_3_)_2_·3H_2_O was dissolved in 15 mL DW. The mixture of 0.3948 of H_3_BTC and 0.0769 g of BA was dissolved in 15 mL absolute ethanol. Then, both solutions were mixed and stirred for 30 min. The resulting mixture and 0.5 g of CF were transferred into a Teflon autoclave and heated in an oven at 110 ℃ for 24 h. The resultant blue fibrous products (HCF) were filtered in vacuum and completely washed by ethanol and DW. Finally, the products were dried at 100 ℃ in vacuum.

In the above preparation process of HCF, by the hydrothermal synthesis method firstly H_3_BTC react with BA in a Teflon autoclave to obtain the MOF product (noted as HST), and then HST composites with the raw material CF to produce HCF.

### Characterization

The structure characteristics of HCF, CF and HST were analysed by Fourier transform infrared (FTIR) spectroscopy (Bruker VERTEX 70, Germany). The morphology characteristics of the samples were obtained on a scanning electron microscope (SEM) (Helios 600i, Japan). X-ray photoelectron spectroscopy (XPS) of HCF and CF were studied using an ESCALAB 250 X-ray photoelectron spectroscope (Thermo fisher, USA). Thermal stability of the products was studied by thermogravimetric analysis (TGA) spectroscopy (Netzsch STA449F5, Germany) from 30 to 900 ℃ at a heating rate of 10 K/min under an argon flow.

### Adsorption studies

The U(VI) stock solutions were prepared by dissolving UO_2_(NO_3_)_2_·6H_2_O in DW; then, small amounts of concentrated HNO_3_ was added to avoid the hydrolysis of UO_2_^2+^. The working U(VI) solutions were prepared by appropriately diluting the stock solutions immediately before their use. The adsorption capacities of U(VI) onto HCF and CF were investigated as a function of the solution pH, contact time, initial U concentration and temperature by batch adsorption experiments. The solution pH was adjusted by adding NaOH and HCl and measured using a glass electrode (Leici PHS-3C, China). HCF or CF was added to 20 mL U(VI) solution and shaken in a shaker (Kangshi, China). After filtration, the U(VI) concentration in the solution was determined by a micro-quantity uranium analyser (MUA model, China). All experiments were performed in triplicate, and the data are presented as the mean values. The removal efficiency (*R* (%)) and adsorption amount of U(VI) on HCF or CF (*q* (mg g^−1^)) of U(VI) in solution were calculated using Eqs. (10) and (11)^59^, respectively.

10 $$R(\% ) = \frac{{c_{0} - c_{t} }}{{c_{0} }} \times 100,$$

11 $$q(mg \cdot g^{ - 1} ) = \frac{{(c_{0} - c_{t} )}}{m} \times V,$$

where *c*_0_ (mg L^−1^) is the initial U(VI) concentration, *c*_*t*_ (mg L^−1^) is the U(VI) concentration at time *t* in the solution, V (L) is the solution volume, and m (g) is the adsorbent mass.

In the desorption experiments, the obtained U(VI)-loaded HCF was washed with DW and rinsed in 3 M HNO_3_ for 24 h; then, it was thoroughly washed in DW until U(VI) ions were not detected in the rinsing solution. The dried and regenerated adsorbent was reused for further adsorption experiments, and this recycling procedure was repeated five times.

## Conclusion

The composite HCF with lower cost was fabricated via a facile solvothermal approach to adsorb U(VI) from aqueous solutions. The preparation cost of the adsorbent was significantly reduced by using BA with lower cost as the ligand to replace part of the traditional ligand H_3_BTC. HCF shows favourable adsorption capacity for U(VI) with maximum adsorption capacities of 241.28 mg g^−1^ at pH 3.0 compared to CF. HCF shows favourable regenerability, and the U(VI) removal percentage was 88.24% after five cycles. This work offers a new and cost-effective adsorbent HCF, which can be effectively used as a promising sorbent to remove U(VI) from the real multi-component U(VI)-containing nuclear waste influents. Furthermore, due to the advantageous fibrous form, HCF can be easily separated from aqueous solutions, which enhances post-treatment efficiency for further practical applications.
